# The Role of Propranolol as a Repurposed Drug in Rare Vascular Diseases

**DOI:** 10.3390/ijms23084217

**Published:** 2022-04-11

**Authors:** Angel M. Cuesta, Eunate Gallardo-Vara, Juan Casado-Vela, Lucía Recio-Poveda, Luisa-María Botella, Virginia Albiñana

**Affiliations:** 1 Departamento de Bioquímica y Biología Molecular, Facultad de Farmacia, Universidad Complutense de Madrid, 28040 Madrid, Spain; angcuest@ucm.es; 2 CIBERER, Centro de Investigación Biomédica en Red de Enfermedades Raras, ISCIII, Unidad 707, 28029 Madrid, Spain; luciarecio@hotmail.com; 3 Yale Cardiovascular Research Center, Department of Internal Medicine, Yale University School of Medicine, 300 George Street, New Haven, CT 06511, USA; eunate.gallardo@yale.edu; 4 Facultad de Ciencias Experimentales, Universidad Francisco de Vitoria, Pozuelo, 28223 Madrid, Spain; jucasado@ing.uc3m.es; 5 Departamento de Bioingeniería, Escuela Politécnica Superior, Universidad Carlos III de Madrid, Av. de la Universidad, 30, 28911 Madrid, Spain; 6 Centro de Investigaciones Biológicas Margaritas Salas, 28040 Madrid, Spain

**Keywords:** beta-adrenergic receptor antagonist, propranolol, HIF, apoptosis, inflammation, angiogenesis

## Abstract

Rare Diseases (RD) are defined by their prevalence in less than 5 in 10,000 of the general population. Considered individually, each RD may seem insignificant, but together they add up to more than 7000 different diseases. Research in RD is not attractive for pharmaceutical companies since it is unlikely to recover development costs for medicines aimed to small numbers of patients. Since most of these diseases are life threatening, this fact underscores the urgent need for treatments. Drug repurposing consists of identifying new uses for approved drugs outside the scope of the original medical indication. It is an alternative option in drug development and represents a viable and risk-managed strategy to develop for RDs. In 2008, the “off label” therapeutic benefits of propranolol were described in the benign tumor Infantile Hemangioma. Propranolol, initially prescribed for high blood pressure, irregular heart rate, essential tremor, and anxiety, has, in the last decade, shown increasing evidence of its antiangiogenic, pro-apoptotic, vasoconstrictor and anti-inflammatory properties in different RDs, including vascular or oncological pathologies. This review highlights the finished and ongoing trials in which propranolol has arisen as a good repurposing drug for improving the health condition in RDs.

## 1. Rare Diseases and Drug Repurposing Opportunities

Rare Diseases (RD) are conditions occurring in less than 5 in 10,000 of the general population. According to the World Health Organization (WHO), approximately 6000–8000 different RDs exist, affecting 400 million people globally, however, less than 5% have an effective treatment [1].

Most of the RDs are life threatening, and the demand for treatments is urgent. However, since development costs of medicines for small numbers of patients may not be recovered, pharmaceutical companies are not primarily focused on RD research [2]. Consequently, drugs to treat RDs are called orphan drugs (OD).

Two main strategies for identifying ODs are currently considered. The first is looking for new medicines, either treatments based on gene therapies or combined gene-cell therapies. This would constitute discovering the drug from the scratch but drug development from the test tube to the patient takes around 10–15 years and the cost has been estimated to rise up to more than 1 billion €. Furthermore, it takes 6–8 years to reach clinical phases and only 1 in 10,000 drug candidates is successful. The second strategy, drug repurposing (or repositioning) (DRP) means establishing new medical uses for already known drugs. This strategy is especially useful in RDs, as a quick and less expensive alternative with an added value of the immediate use in clinical trials since safety is known from the first indication. Currently, about 20% of the ODs are repurposed [3,4].

Regulatory agencies such as the European Medicines Agency (EMA) and the North American Food and Drug Administration (FDA) have tried to encourage research into these disorders by tax waiving, fast-track approval, grants and fee waivers [5]. Interestingly, some of the commercial barriers to drug repurposing, for example concerns regarding off-patent drugs, are not as important when addressing neglected conditions since therapeutic research for such disorders are not driven by profit motivation. Repurposing a low-cost off-patent drug is ideal for ensuring a prompt patient accessibility.

## 2. Propranolol as a Repurposed Drug for Rare Diseases

Propranolol, a non-specific β1-and β2-adrenergic receptor (ADRB1-2) antagonist, initially prescribed for cardiac disorders, has, in the last 10–15 years, become a paradigmatic example of an extremely valuable drug, showing multiple off-target clinical therapeutical properties in cancer and RDs.

Propranolol was initially proposed in 1964 as a β-receptor antagonist that showed suitable properties to fight against cardiac disorders [6], acting as a nonselective β1- and β2-adrenergic antagonist without partial agonist effects. Since then, the treatment of cardiac disorders has remained the main clinical prescription for propranolol [6].

Nevertheless, propranolol continued with the initial therapeutic indication until 2008 when Léauté-Labrèze et al., based on the induction of apoptosis in capillary endothelial cells (ECs) [7], showed its clinical antiangiogenic therapeutic properties in 11 cases of Infantile Hemangioma (IH) [8]. IH is a benign vascular tumor present in a 4–5% of neonates and normally affecting the face and limbs. When IHs do not spontaneously remit, surgery was the only treatment before the advent of propranolol. Several case reports and successful trials later, Hemangiol (an oral liquid presentation of propranolol), was designated for the IH by EMA in 2014 [9,10,11].

After the success of IH treatment with propranolol, its use and potential therapy has been expanded to other RDs such as Hereditary Hemorrhagic Telangiectasia (HHT), von Hippel-Lindau disease (VHL), soft tissue sarcoma, Cerebral Cavernous Malformations (CCMs), and Lafora disease [12,13,14,15]. More recently, in a murine in vivo model of Lafora, a neurological RD where propranolol shows a potential therapeutic effectiveness, modulating the microglia and astroglia inflammation, being proposed as a novel treatment [16].

**Figure 1:**
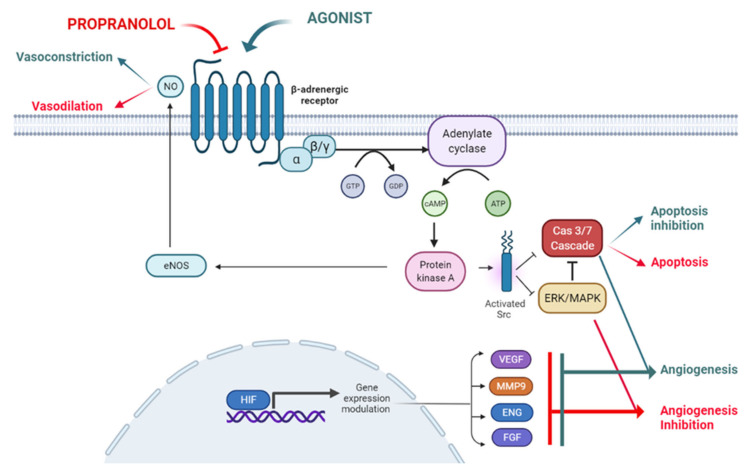
The ADBR signaling in the presence of the ligand, which will be blocked by propranolol. According to this model propranolol would decrease de adenylate cyclase activity, decreasing the cAMP (cyclic Adenosine Monophosphate) levels and the activation of PKA (Protein Kinase A). Activation of eNOS (endothelial Nitric-Oxyde Synthase) by PKA will be decreased leading to vasoconstriction. On the other hand, the decrease in PKA activity will affect Src (Proto-oncogene tyrosine-protein kinase Src) expression impairing the HIF-1 (Hypoxia Inducible Factor 1) nuclear translocation with the downregulation of its nuclear targets such as pro-angiogenic genes VEGF (Vascular Endothelial Growth Factor), MMP9 (Matrix Metallopeptidase 9), ENG (Endoglin) and FGF (Fibroblast Growth Factor). The decreased phosphorylation of ERK/MAPK (ERK, Extracellular Signal-Regulated Kinase; MAPK, Mitogen-Activated Protein Kinase) kinases cascade and Src will activate the caspase cascade leading to apoptosis. Created by Biorender.com.

Propranolol exhibits certain lipophilic properties which makes it able to cross the blood brain barrier [17]. It also displays pharmacodynamic characteristics such as vasoconstriction, inhibition of angiogenesis and induction of apoptosis, all of which are related to its therapeutic possibilities, as described below and depicted in Figure 1.

### 2.1. Vasoconstriction

Propranolol induces vasoconstriction by counteracting adrenalin effects. Adrenalin acts through Adrenoreceptor beta 2 (ADRB2), a G protein-coupled receptor (GPCR) which activates adenylate cyclase (AC) and converts ATP into cyclic adenosine monophosphate (cAMP). This cAMP diffuses to the cytosol and activates cAMP-dependent protein kinase A (PKA). PKA can, in turn, phosphorylate intracellular proteins and thus modulate their activity. This signaling pathway triggers vasodilation in pericytes and smooth muscle cells [18]. Contrary to this adrenaline effect, propranolol, leading to vasoconstriction, reduces blood flow in the vessels and capillaries which feed the tumor and therefore, and impairs the tumor growth [19] (Figure 1).

### 2.2. Inhibition of Angiogenesis

Under physiological conditions, angiogenesis is tightly controlled [20], not as in pathological conditions such as cancer [21]. VEGF, the main proangiogenic factor, is regulated by HIF. VEGF is secreted and diffuses into the surrounding tissue, inducing proliferation of adjacent ECs and coordinating differentiation of vascular cells (ECs, smooth muscle cells and pericytes) into forming functional vessels (angiogenesis), improving local oxygen supply (Figure 1) [22,23]. The anti-angiogenic effect of propranolol is shown in IH [8] and in vitro with the reduction in Hypoxia Inducible Factor (HIF) levels and vascular endothelial growth factor (VEGF) secretion and thus impairment of the tumor-related angiogenic process [24,25,26,27].

### 2.3. Induction of Apoptosis

Blockade of ADRBs by propranolol can induce apoptosis in different types of cells and tumors in vitro, e.g., in ECs [7].

The authors hypothesize that β-adrenergic antagonists counteract the Src-MAPK-mediated inhibition of apoptosis caused by β-adrenergic agonists, resulting in an increased rate of apoptosis. More recently, we have shown that propranolol induces apoptosis in in vitro cultures of HeLa cells, renal carcinoma cell lines and Central Nervous System-hemangioblastomas (CNS-HB) and renal carcinoma primary tumors from von Hippel-Lindau (VHL) patients [25,26,28] (Figure 1).

## 3. Propranolol for a Benign Vascular Tumor (IH)

Since the already mentioned breakthrough of propranolol for IH in 2008 [8], 25 different clinical trials have been registered in EU, USA, and Australia/New Zealand (Table S1). IH is not a rare vascular tumor in a strict sense, but it was the use of propranolol for IH which triggered the discovery of propranolol’s additional therapeutic properties and allowed its use on real RDs. The results of the trials, mainly conducted in newborns, almost invariably have shown the success of propranolol in decreasing or eliminating IH. In these trials, propranolol was used at different doses; from 1 to 3 mg/kg body weight/day for 3 to 12 months. The most remarkable ones are commented herewith. The phase 2 trial (ACTRN12611000004965) and the phase 2/3 (NCT00744185) and (NCT01056341), showed success in treated groups. Propranolol reduced the volume, color, and IH thickness. The known adverse events associated with propranolol (hypoglycemia, hypotension, bradycardia, and bronchospasm) occurred infrequently with no significant differences to placebo groups [11,29,30].

Finally, a single arm, phase 3, multinational study (EudraCT Number: 2014-005555-80) was conducted in patients aged between 35 and 150 days with high-risk IH [31]. The conclusions for the trial were that long-term propranolol administration significantly increased the success rate in high-risk IH. Efficacy was sustained in most patients up to 3 months after stopping treatment. Retreatment was effective and the safety profile satisfactory. Following these results with relatively mild side effects, EMA authorized, in 2014, the designation of Hemangiol as a new commercial presentation of propranolol (oral liquid presentation of propranolol adapted for infants) for the treatment of IH.

## 4. Propranolol in Vascular and Tumoral Rare Diseases

### 4.1. Hereditary Hemorrhagic Telangiectasia (HHT)

HHT is a vascular RD caused by mutations in *ENG* and *ALK1* genes in more than 90% of patients. The prevalence is 1:5000 and characterized by the presence of epistaxis (nosebleeds), mucocutaneus telangiectasias and arteriovenous malformations in organs such as lungs, brain and liver [32,33,34]. Propranolol and timolol (another non-selective beta-adrenergic blocker) have shown antiangiogenic properties related to vasoconstriction, inhibition of EC migration and proliferation, and reduced VEGF expression [8,9,10,35]. Since excess of VEGF is involved in the development of telangiectasias, the properties of these non-selective adrenergic β-blockers may be considered a potential treatment option for HHT patients. According to several reports, topical timolol (0.5% ophthalmic solution) decreased the frequency and severity of epistaxis [36,37].

Timolol has also proved to be efficient at lower doses (0.1% timogel) in decreasing the extension and appearance of mucocutaneous telangiectasias in HHT, showing 100% and 75% improvement in HHT2 and HHT1 patients, respectively [38].

Clinical studies support the reduction in nosebleeds in HHT patients after propranolol nasal topical administration. In a pilot study with 6 patients, 1.5% propranolol gel was applied at a dose of 0.5 mL/day per nostril. The severity of epistaxis and the number of blood transfusions pre- and post-administration were reduced rapidly and significantly [39]. As continuation of this study, the same group successfully finished a recent double-blind placebo-controlled study to assess the efficacy and safety of topical propranolol for moderate–severe epistaxis in 24 HHT patients [40].

The combination of sclerotherapy with 1% polydocanol and the use of propranolol cream at 0.5%, prepared in a hospital pharmacy, was evaluated in a cross-sectional study of 38 HHT patients. This combined therapy significantly reduced the frequency and severity of epistaxis, with a highly significant improvement in the Epistaxis Severity Score (ESS), accompanied by a significant increase in the quality of life of these patients [41].

Since systemic propranolol has the potential side effects of bradycardia and hypotension, propranolol could be used in a systemic way in HHT patients with hypertension. A clinical study with oral propranolol (40–120 mg/day) was conducted in 7 hypertensive HHT patients. Among them, HHT epistaxis disappeared completely in five out of seven, while in the other two the bleeding reduction was highly significant [42].

### 4.2. Von Hippel-Lindau Disease (VHL)

Inspired by the work of Leauté-Labrèze [8], our lab demonstrated in vitro the therapeutic properties of propranolol in the VHL RD. VHL is an autosomal dominant inherited syndrome (1:36,000 births) [43,44] whose patients are heterozygous for mutations in the *VHL* tumor suppressor gene (3p25–p26). The VHL protein (pVHL) is responsible of labeling for proteasomal degradation of HIF-1 under normoxic conditions. Nevertheless, mutation, inactivation, or loss of the second allele of *VHL* (loss of heterozygosity), leads to a null or non-functional pVHL [45,46]. In the absence of functional pVHL, HIF-1 is not degraded and translocates to the nucleus, triggering the hypoxia program, normally silent in normoxia [47,48]. The type of tumors are mainly retinal -or CNS-HB, brain stem and spinal cord, as well as clear cell renal cell carcinoma (ccRCC) and endocrine tumors: pheochromocytoma, and pancreatic islet tumor. Endolymphatic sac tumors and cysts in testes and broad ligament are other manifestations of VHL [49,50].

Since surgery remains the only therapeutic option and those repeated surgeries decrease patients’ quality of life [51], VHL patients demand an effective drug that might halt the tumor progression or delay surgical procedures.

Several trials for VHL have followed the common strategies in cancer chemotherapy, such as antiangiogenic molecules such as bevacizumab or protein tyrosine kinases inhibitors (TKI) such as sunitinib but have shown limited or no response in VHL tumors [49]. Recently and only for non-metastatic ccRCCs, the selective HIF-2α inhibitor belzutifan was tested in 61 VHL patients. The data showed a limited response, as none of the patients showed complete response and 28 showed partial response. In addition, some responses were observed in CNS, retinal, and pancreatic lesions [52,53].

An alternative in the field of angiogenesis is represented by ADRB-blockers. Briefly, propranolol treatment of primary cultured HB cells decreased viability, increased apoptotic death, and downregulated HIF-1 protein expression levels. Concomitantly, HIF-1 targets such as VEGF, EPO, and SOX appeared downregulated at RNA and protein levels [24].

Furthermore, in a phase 3 trial (EudraCT: 2014-003671-30) (Table 1) [25,28] in which 7 VHL patients bearing multiple retinal HBs were treated with oral propranolol (120 mg/day for 12 months), neither the size nor the number of HBs increased, 2 retinal exudates disappeared, and plasma levels of VEGF and miR210 (a direct HIF target) were reduced in all the 7 patients [28]. As side effects, driven by its ADRB1-blockade, hypotension and bradycardia were reported. These findings aided the OD designation, in 2017, for propranolol hydrochloride for the treatment of VHL by EMA (EU/3/17/1841). In addition, prospective analysis of propranolol administered in an off-label use on ccRCC-VHL tumor growth kinetics in VHL patients showed a stabilization of RCCs size during propranolol treatment (from 15 to 47 months), as it has also been observed in the head and neck, esophageal, stomach, colon, and prostate cancers [54].

**Figure 2:**
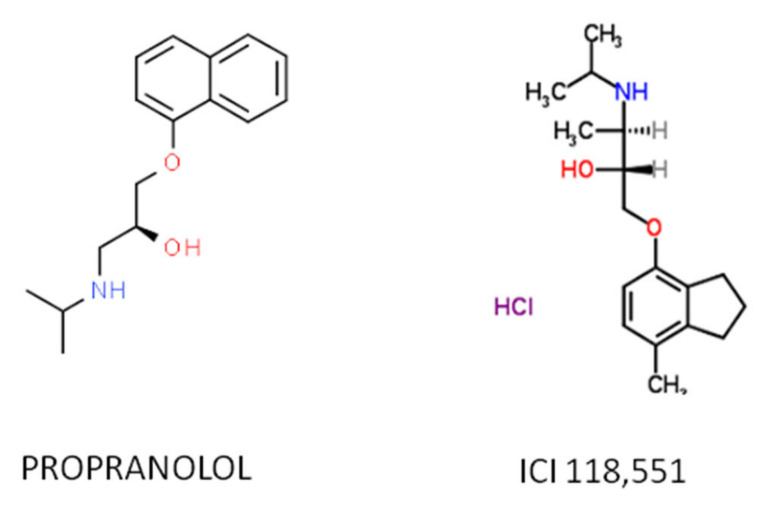
The atomic structure of the propranolol and ICI 118,551 molecules.

Recent work has demonstrated that the therapeutic effect of propranolol is mainly due to the ADRB2-blockade, hence the ideal therapeutic drug would be a specific ADRB2 blocker. Among the ADRB2-blockers, the selective antagonist erythro-D,L-1(methylinden-4-yloxy)-3-isopropylaminobutan-2-ol, known as ICI-118,551, has recently shown therapeutic properties similar as those of propranolol in vitro and in vivo and shows a much higher affinity for ADRB2 than propranolol [55,56,57], making it a potential substitute (Figure 2).

Propranolol and ICI-118,551 triggered similar gene expression changes in VHL primary cultures. To highlight, the induction of apoptosis through upregulation of pro-apoptotic genes such as *BAX*, *CASP3*, *7* and *9*, and the downregulation of HIF target genes such as *VEGF*, *SOX2*, *OCT4*, *E*PO, and *AQP-1* [27]. *AQP-1* is particularly interesting as, under HIF induction, it encodes a transmembrane water channel protein [58,59,60] that allows increased fluid flow across the cell membrane and increased number and size of cystic growths, commonly described in VHL tumors and leading to early stages of inflammatory processes and subsequent cancer development [61,62,63].

### 4.3. Paraganglioma Syndrome (PPGL)

Paraganglioma syndrome (PPGL) is another rare tumor (11:100,000 births), driven by mutations in *SDH* *A*, *B*, *C* (succinate dehydrogenase genes) and lacks specific treatment but surgical resection [64]. Symptomatology, being related to catecholamine hypersecretion, is usually hypertensive or paroxysmal [64], but it also develops symmetrically distributed tumors along the paravertebral, of neural crest origin, as well as gastrointestinal tumors, renal cancer, bone metastasis and pituitary adenomas [65]. In 2007, a case report described how the combination of high doses of propranolol (3 mg/kg/day) and temozolomide (alkylating agent) successfully reduced the number and size of the metastases even after 7 months of treatment withdrawal [65].

**Table 1:** Propranolol in single or combined therapy in clinical trials of rare diseases.

Trial ID	Study Title	Status	Conditions	Propranolol Compared with	Outcome Measures	Phase	N	Start Date	Results
NCT01058317	Propranolol Administration in Pediatric Patients With Recurrent Respiratory Papillomatosis	W	Recurrent Respiratory Papillomatosis	-	Number of surgeries Improved voice quality	2/3	0	2010	[66]
2014-003671-30	Therapeutic effect of propranolol in a series of patients with von Hippel-Lindau disease and retinal hemangioblastomas in short, medium and long term treatment.	C	Retinal Hemangioma	-	Number and size of the retinal or CNS hemangioblastomas	3	10	2014	[25,28]
2015-005177-21	Dose-Finding of Propranolol in combination with metronomic fixed oral cyclophosphamide based on Bivariate efficacy-tolerability outcome in patients with locally advanced or metastatic angiosarcoma	O	Angiosarcoma	cyclophosphamide	Toxicity and Response rate: Progression. Free survival. Growth modulation index. Overall survival. Tolerability.	1/2	24	2015	-
NCT02732678	Dose-Finding of Propranolol in Combination With Metronomic Fixed Oral Cyclophosphamide Based on Bivariate Efficacy-tolerability Outcome in Patients With Locally Advanced or Metastatic Angiosarcoma: A Collaborative and Innovative Phase I-II Sequential Trial by the French Sarcoma Group (GSF/GETO)	U	Angiosarcoma	propranolol	Toxicity of each tested propranolol dose level in association to cyclophosphamide assessed according to NCI-CTC AE Version 4.0. Non-progression rate.	1/2	24	2016	-
NCT03633747	Efficacy Evaluation of Propranolol Treatment of Hepatic Hemangioma	R	Hemangioma Liver	-	Tumor size. Objective response rate.	1/2	25	2018	-
NCT03474614	Effect of Oral Propranolol on mRNA Expresssion in Symptomatic Cavernous Malformation	NYR	Cerebral Cavernous Malformations	-	Global mRNA and miRNA expression in the blood and tissue. Adverse event.	2	20	2018	-
NCT03589014	Treat_CCM: Propranolol in Cerebral Cavernous Malformation	R	Cerebral Cavernous Malformations	-	Adverse clinical events and outcomes. De novo CCM lesions depiction. CCM size. Micro-hemorrhages at MRI.	2	70	2018	[67]
NCT03523650	Oral Propanolol for Surgically Inaccessible Cavernous Malformations	U	Cavernous Malformations Cerebral and/or Spinal	Propranolol placebo	Number of symptomatic and silent hemorrhages on MRI. Rate of de novo lesion formation; changes in rate of breakthrough seizures or other neurological deficits	1	346	2018	-
NCT04518124	Propranolol in Angiosarcoma	R	Angiosarcoma	-	Clinical and Histological response.	2	14	2019	[68]
2019-002947-41	Neoadjuvant trial on the efficacy of propranolol monotherapy in angiosarcoma (PROPANGIO)	O	Angiosarcoma	-	Tumor size examination, according to RECIST criteria. Difference in proliferation index.	2	28	2019	[68]
NCT04406870	Sirolimus in the Treatment for Infantile Hepatic Hemangioendothelioma(IEEH)	NYR	Hemangioendothelioma of Liver	sirolimus	Changes on tumor size, PIVKA-II and alpha-1 fetoprotein (AFP)	4	36	2020	[69]

Compilation of the interventional clinical trials registered at the EU Clinical Trials Register (https://www.clinicaltrialsregister.eu, accessed on 24 March 2022) and in the U.S. National Library of Medicine (https://clinicaltrials.gov, accessed on 24 March 2022). Status: C (Completed); NYR (Not yet recruiting); R (Recruiting); O (Ongoing); U (Unknown); W (withdrawn).

### 4.4. Cerebral Cavernous Malformations (CCMs)

Cerebral cavernous malformations (CCMs) are vascular malformations characterized by clusters of enlarged permeable capillaries in the CNS (prevalence 1:200–1000 individuals) [70]. They may result in intracranial hemorrhages, epileptic seizure(s), or focal neurological deficits, and can cause severe disability. Worldwide, CCMs represent the second most common intracranial vascular malformation in humans, and their familial form accounts for one-fifth of cases. Neurosurgical excision, and perhaps stereotactic radiosurgery, are the only therapeutic options available. Some case reports suggested that propranolol might modify disease progression [71,72] and contributed to the initiation, in 2008, of three different trials administering propranolol as monotherapy. These trials are still ongoing and whose results have not been posted or published yet. Two pre-clinical studies of propranolol have shown reduced lesion burden upon propranolol treatment [73,74], including vascular stabilization with reduced leakage [74].

The phase 1 “Oral propranolol for Surgically Inaccessible Cavernous Malformations” (NCT03523650) [75], pursues the number of symptomatic and silent hemorrhages on MRI, the rate of de novo lesion formation, and the changes in rate of breakthrough seizures or other neurological deficits (Table 1).

The phase 2 “Effect of Oral propranolol on mRNA Expression in Symptomatic Cavernous Malformation monotherapy” (NCT03474614) [76], focuses on measuring the effects of low-dose oral propranolol (60 mg/day) on global mRNA and miRNA expression in the blood and tissues of the treated patients Table 1.

The phase 2 “Treat-CCM: Propranolol in Cerebral Cavernous Malformation” (NCT03589014) [67], will analyze clinical CCM-related events (i.e., FND, functional neurological disorder or ICH, intra-cerebral hemorrhage), circulating biomarkers and miRNA, de novo lesions depiction on MRI, allocation, and MRI signal characteristics of CCM lesions (diameter and length) at MRI, micro-hemorrhages according to MRI, and dynamic contrast enhanced permeability (DCEP) at MRI (Table 1).

### 4.5. Angiosarcoma

Angiosarcoma, a subtype of soft tissue sarcoma, is an ultra-rare and aggressive malignant tumor (1:1,000,000 births, according to NIH data) with a high metastatic potential and recurrence rate. Despite optimal treatment with surgery, with or without radiation, the prognosis remains poor. Inspired by the results of propranolol in IH, a small scale of individual angiosarcoma cases were successfully treated with propranolol, leading to OD designation for angiosarcomas and soft tissue sarcoma in 2016 (EU/3/16/1805).

Two very similar phase 2 clinical trials, in the EU (EudraCT: 2019-002947-41) and in the USA (NCT04518124), developed by the Netherlands Cancer Institute, are active and ongoing during the writing of this review [68]. The clinical response to oral propranolol as monotherapy in patients with angiosarcoma will be evaluated as primary endpoint and histological response as secondary endpoint (Ki-67 as biomarker). The study will be considered positive when at least three patients respond to the propranolol.

### 4.6. Tuberous Sclerosis

A mutation in the mTOR inhibitors TSC1*-2* (1:5000–10,000 births) triggers this life threatening rare neurocutaneous disorder characterized by multisystem hamartomas, usually affecting the brain, eye, skin, heart, kidney, and lung, and is also associated with neuropsychiatric disorders. This multisystemic disease is treated with supportive and symptomatic care. Based on its unregulated cell proliferation and disproportionate glutamate activity, propranolol has been thought to induce regression or stabilization of the generated renal angiomyolipoma. Hence, the phase 2 trial NCT02104011 completed in 2018. Unfortunately, no information is yet available [77] (Table 1).

### 4.7. Other Rare Carcinomas

It is worth mentioning that propranolol was also registered in a clinical trial with pediatric patients affected by Recurrent Respiratory Papillomatosis, with positive results, improving voice quality and decreasing the number of surgeries [66]. Finally, within the group of RDs, propranolol (alone or in combination with sirolimus) was tested for hemangioendothelioma of liver (NCT04406870). The results showed that propranolol was as effective as sirolimus and that no serious adverse reactions were observed [69].

## 5. Conclusions

- Propranolol (an antagonist of ADBR1-2) has emerged as a candidate repurposed drug for an increasing number of RDs. It is a well-characterized drug (both in vitro and in vivo), with a safety profile and therapeutic experience that sufficiently support its use in mono- or combination therapies, as a repurposing drug in spite of the well-known side effects mentioned earlier: bradycardia and hypotension of repurposing drug in clinical application [78];
- Propranolol is involved in a range of physiological and molecular mechanisms that support its potential therapeutic value, including vasodilation, apoptosis of cells in active division by increase in Bax, and Caspases3/7, inhibition of MAPKs, downregulation of HIF in hypoxic or pseudohypoxic processes, antiangiogenic drug decreasing VEGF and EPO protein expression (both HIF targets), and inhibition of dedifferentiating genes such as *SOX-2*;
- Subsequently to propranolol success for IH treatment, propranolol has been used in monotherapy and combination therapy in different clinical trials for different tumor types, including RDs;
- In RDs, the designation of propranolol as an OD for the treatment of VHL was a remarkable fact. In angiosarcoma, propranolol has been successful in treating a small number of patients, and further trials are ongoing. In CCM, three parallel clinical trials are currently underway.

## Data Availability

Data supporting reported results can be found at the EU Clinical Trials Register (https://www.clinicaltrialsregister.eu, (accessed on 24 March 2022)) and in the U.S. National Library of Medicine (https://clinicaltrials.gov, (accessed on 24 March 2022)).

## Supplementary Materials

The following supporting information can be downloaded at: https://www.mdpi.com/article/10.3390/ijms23084217/s1.
