# Supplementary material for: The Role of Propranolol as a Repurposed Drug in Rare Vascular Diseases
Source: Int J Mol Sci. 2022 Apr 11;23(8):4217. doi: 10.3390/ijms23084217 (PMC9025921; doi:10.3390/ijms23084217)
Supplement: Supplementary file 1 [file ijms-23-04217-s001.zip › Table S1.pdf]

Supplementary Materials

Supplementary Table S1. **Propranolol in single therapy clinical trials.** Compilation of the interventional clinical trials registered at the EU Clinical Trials Register (https://www.clinicaltrialsregister.eu), the U.S. National Library of Medicine (https://clinicaltrials.gov), and the Australian New Zealand Clinical Trials Registry (http://www.anzctr.org.au/Default.aspx). Only interventional trials (with results or not finished), whether propranolol as single therapy has been tested, are listed.

Trial ID	Study Title	Status	Conditions	Propranolol compared with	Outcome Measures	Phase	N	Start Date
NCT00744185	Propranolol in Capillary Hemangiomas	T	Hemangioma Capillary	placebo	Size and thickness variation.	2/3	14	2008
2008-000202-36	Randomized clinical trial of propranolol versus placebo in the treatment of pediatric capillary hemangiomas	O	Capillary hemangiomas	placebo	Evolution of hemangioma thickness at one month of treatment in infants.	3	50	2008
NCT01211080	Off Label Use of Propranolol for Infancy Hemangiomas	C	Hemangioma	propranolol	Cosmesis of the lesion and surrounding skin.<br>Side effects.<br>Hemangioma size.		72	2008
ACTRN12611000004965	Propranolol for infantile haemangiomas	C	Infantile hemangioma	-	Change in volume.	2	40	2009
NCT00967226	Propranolol Versus Prednisolone for Treatment of Symptomatic Hemangiomas	T	Hemangioma of Infancy	prednisolone	Size of Hemangioma.<br>Tolerability of Medication.<br>Number of Serious Adverse Events.	2	19	2009
2009-017241-55	Open randomized study on propranolol vs prednisone for haemangioma in patients younger than 18 months of age	S	Hemangiomas	propranolol prednisona	More or equal efficacy of propranolol, with less side effects than prednisone for haemangiomas.	3	18	2009
NCT01056341	Study to Demonstrate the Efficacy and Safety of Propranolol Oral Solution in Infants With Proliferating Infantile Hemangiomas Requiring Systemic Therapy	C	Infantile Hemangioma	placebo	Complete or Nearly Complete Resolution at Week 24.	2/3	512	2010
2009-014571-49	The use of systemic propranolol in congenital hemangiomas	O	Congenital Hemangioma	-	Efficacy and tolerability during the first months of life.	2	10	2010
2009-018102-22	A multicentre, open-label, repeated-dose, pharmacokinetic study of	C	Infantile Hemangioma	-	Pharmacokinetic assessment (rate and extent of absorption) in	1	18	2010

	Propranolol in infants treated for proliferating infantile hemangiomas (IHs) requiring systemic therapy				plasma concentrations.			
2010-019754-41	Multicenter, prospective, single-arm phase III study of the efficacy and safety of oral therapy with propranolol (ProAngiol juice; 2 mg/kg bw/d with optional dose increase to 3 mg/kg bw/d) in infants 4 weeks to 11 months of age with severe proliferating hemangiomas	C	Hemangioma requiring systemic therapy	-	Proliferation, depth and body size. Blood pressure, heart rate change and blood glucose change. Side effects.	3	60	2010
NCT01072045	Comparative Study of the Use of Beta Blocker and Oral Corticosteroid in the Treatment of Infantile Hemangioma	C	Hemangioma	propranolol, prednisone	Reduction on tumor volume, based on direct measurement and photographic analysis.	2	50	2010
NCT01074437	Corticosteroids With Placebo Versus Corticosteroids With Propranolol Treatment of Infantile Hemangiomas (IH)	T	Hemangioma	propranolol, prednisolone , placebo	Changes in IH Size and Vascularity. Lesion Regression.	2	9	2010
NCT04651049	Systemic Propranolol for the Treatment of Paediatric Patients With Infantile Hemangiomas	C	Infantile Haemangiomas	propranolol	Change on the height or weight of the patients.		128	2010
2011-003144-50	A randomised, double blind, controlled, multicentre study in infants with infantile hemangioma to compare propranolol gel to placebo	C	Infantile Hemangioma	placebo	Efficacy, safety profile and local tolerance.	2	80	2011
ACTRN1261200 1227886	Renin-Angiotensin System and Other Markers in Strawberry Birthmarks in infants and young children with growing problematic haemangiomas	W	Hemangioma	-	Serum ACE, Angiotensin II, alpha-feto protein and Renin Levels.	NA	40	2012
NCT01512173	Study in Infants With Infantile Hemangioma to Compare Propranolol Gel to Placebo	C	Infantile Hemangioma	propranolol gel placebo	Complete/nearly complete resolution of the Infantile Hemangioma at week 12. Persistence of efficacy 12 weeks after the end of treatment. Local tolerance of the propranolol gel	2	82	2012
NCT01743885	Efficacy and Safety of Propranolol Versus Acebutolol on the	T	Hemangioma	propanolol acebutolol	Hemangioma size. Tolerance.	3	55	2012

	Proliferative Phase of Infantile Hemangioma				Proportion of patients requiring treatment with corticosteroids.			
NCT01908972	The Safety and Efficiency of Propranolol as an Initial Treatment for Pediatric Hemangioma	C	Hemangioma	prednisolone	Clinical Response.<br>Percent Reduction.<br>Change in Color.<br>Size Reduction of Ulceration.<br>Reepithelization.<br>Regression.<br>Systolic Blood Pressure.<br>Glucose Levels.<br>Facial Edema.<br>Gastroesophageal Reflux.<br>Adverse Drug Reaction.	4	34	2013
NCT02342275	Efficacy and Safety of Propranolol Versus Atenolol on the Proliferative Phase of Infantile Hemangioma	C	Hemangioma	atenolol	Color and size of IH.<br>Frequency of adverse events.<br>Cardiovascular examinations.<br>Blood glucose.<br>Neurodevelopment.<br>Quality of life.	3	377	2013
2014-005555-80	Efficacy and safety of Hemangiol solution in the treatment of high risk infantile hemangioma. A Multinational Single Arm Study	C	Infantile Hemangioma	-	Efficacy (6 to 12 months of age).<br>Safety (3 months after interruption).<br>Efficacy of re-administered (6 months in relapse cases).	3	45	2015
NCT02505971	Nadolol Versus Propranolol in Children With Infantile Hemangiomas	C	Infantile Hemangioma	propranolol<br>nadolol	The change in the bulk and color of the hemangioma (IH) at Week 24 compared to baseline.<br>Percent change in IH bulk.<br>Time and dose to reach the 50%, 75% and 100% tumor shrinkage.<br>Inter-rater reliability of the VAS scores.<br>Percentage of patients achieving functional correction.<br>Percent change in the volumetric changes of hemangioma.<br>Percentage of patients with residual changes.<br>Frequency of observed and	3	74	2015

					reported adverse events.			
NCT03237637	Comparative Study to Evaluate the Effectiveness of Atenolol and Propranolol in the Treatment of Infantile Hemangiomas	U	Infantile Hemangioma	propranolol atenolol	Mean difference in number of patients achieving complete clinical clearance of lesions. Mean difference in number of days required to achieve complete clinical clearance of lesion. Mean difference in Hemangioma Activity Score. Frequency of adverse effects. Mean difference in HIF-1a levels before and after treatment.	3	60	2017
NCT04105517	Hemangiol, Post Marketing Surveillance Study	C	Infantile Hemangioma	-	Adverse events. Regression. Diagnosed heart diseases.	NA	500	2019
NCT04288700	Evaluation of the Efficacy of Captopril Versus Propranolol and Timolol as a Treatment of Infantile Capillary Hemangioma	R	Infantile Hemangioma	captopril timolol maleate	Serum levels of VEGF, CD 133 and size of the lesion.	4	100	2019
NCT04684667	'Efficacy of Propranolol in the Treatment of Infantile Hemangioma	NYR	Infantile Hemangioma	-	Complete clinical clearance or volume reduction.	2	100	2021

Phase: NA (Not Applicable). Status: C (Completed); NYR (Not yet recruiting); R (Recruiting); O (Ongoing); T (Terminated); S (Suspended); U (Unknown); W (withdrawn).
